# Adverse Husbandry of Maraena Whitefish Directs the Immune System to Increase Mobilization of Myeloid Cells and Proinflammatory Responses

**DOI:** 10.3389/fimmu.2016.00631

**Published:** 2016-12-23

**Authors:** Tomáš Korytář, Mareen Nipkow, Simone Altmann, Tom Goldammer, Bernd Köllner, Alexander Rebl

**Affiliations:** ^1^Institute of Immunology, Friedrich-Loeffler-Institut, Federal Research Institute for Animal Health, Greifswald-Insel Riems, Germany; ^2^Department of Pathobiology, School of Veterinary Medicine, University of Pennsylvania, Philadelphia, PA, USA; ^3^Fish Genetics Unit, Institute for Genome Biology, Leibniz Institute for Farm Animal Biology (FBN), Dummerstorf, Germany

**Keywords:** interleukins, leukocytes, lower vertebrates, salmonid fish, stocking density, transcriptome, welfare

## Abstract

Adverse life circumstances evoke a common “conserved transcriptional response to adversity” (CTRA) in mammalian leukocytes. To investigate whether this pattern is preserved in lower vertebrates, maraena whitefish (*Coregonus maraena*) were exposed for 9 days to different stocking densities: ~10 kg/m^3^ (low density), ~33 kg/m^3^ (moderate), ~60 kg/m^3^ (elevated), and ~100 kg/m^3^ (high). Transcriptome profiling in the liver and kidney of individuals from each group suggested that crowding conditions activate stress-related signaling and effector pathways. Remarkably, about one-quarter of the genes differentially expressed under crowding conditions were involved in the activation of immune pathways such as *acute-phase response* and *interleukin*/*TNF signaling* attended by the simultaneous reduction of antiviral potency. Network analysis confirmed the complex interdigitation of immune- and stress-relevant pathways with interleukin-1 playing a central role. Antibody-based techniques revealed remarkable changes in the blood composition of whitefish and demonstrated the correlation between increasing stocking densities and elevated number of myeloid cells together with the increased phagocytic activity of peripheral blood leukocytes. In line with current studies in mammals, we conclude that crowding stress triggers in whitefish hallmarks of a CTRA, indicating that the stress-induced molecular mechanisms regulating the immune responses not only are conserved within mammals but were established earlier in evolution.

## Introduction

Fish farming preserves natural resources. However, adverse housing conditions including practice activities, such as selection, handling, transport, (mal-)nutrition, and/or inadequate stocking densities, threaten fish well-being (1–8). In favor of profitability, farmed fish are often kept at densities a 1,000-fold higher than under natural conditions (9, 10). Inadequate stocking densities not only are detrimental to production-related traits such as growth rate (11) but also decrease food uptake (12), increase the level of stress hormones, and thus lead to higher susceptibility to infectious diseases (13–18). Besides influencing the level of the stress hormone cortisol (19–21), stress is known to modulate the hemolytic and agglutinating activity of the serum (22). Furthermore, it affects the phagocytic and complement activities (23), influences the expression of immune-related genes (24), and reduces the humoral response following vaccination (25). However, little is known about the molecular mechanism underlying these physiological changes.

In mammals, adverse life circumstances have been shown to induce a “conserved transcriptional response to adversity” (CTRA), which typically leads to an increased expression of proinflammatory genes and a decreased expression of genes involved in innate antiviral responses (26, 27). These effects stem in part from the increased hematopoietic production of myeloid lineage immune cells (28, 29). Stress has been proven to cause a substantial number of the aforementioned physiological changes in fish, and it is quite conceivable that the mechanisms of a CTRA were established earlier in the evolution, although this has not been investigated in teleost model species yet. Drawing on the example of two killifish populations, it has been demonstrated that sensitivity to stressors—including high stocking densities—may differ between populations depending on their evolutionary history (30). Because of generations of domestication and adaptation, the stress responsiveness of rainbow trout, a salmonid fish, is known to be low (31, 32). By contrast, adaptation to stress conditions may be low in newly established aquaculture species, such as maraena whitefish (*Coregonus maraena* L.), making this salmonid fish an excellent model for the investigation of sensitivity to crowding stress and its impact on whitefish physiology.

The key objective of the present study was thus to identify surrogate markers for well-being, and—particularly—to assess the health status of maraena whitefish. Using a combination of transcriptomic and immunological techniques, we aimed at characterizing the impact of crowding stress on the hallmarks of a CTRA in the liver and kidney as the predominant teleost tissues involved in the stress response (33). Furthermore, based on these findings, we evaluated the immune competence of stressed fish in an *in vitro* experiment with inactivated and viable *Aeromonas salmonicida*, a major threat in whitefish farming (34, 35).

## Materials and Methods

### Fish and Stocking Density Experiment

The stocking density (SD) experiments were performed in water recirculation tanks at the Institute for Fisheries in Born, Germany. Maraena whitefish were aged 205 days post-hatch at the start of the experiments. These were performed in duplicate at four different stocking densities: low density (LD, ~10 kg/m^3^, 33 and 34 individuals), moderate density (MD, ~33 kg/m^3^, 101 and 103 individuals), elevated density (ED, ~60 kg/m^3^, 181 and 185 individuals), and high density (HD, ~100 kg/m^3^, 305 and 309 individuals). Fish were randomly assigned to identical 300-l glass tanks (0.74 m length × 0.58 m width × 0.72 m height) at a 12 h day/night light period. Tanks received brackish water from the Darss-Zingst Bodden Chain (2.5–6 practical salinity units) to the recirculating aquaculture system with an exchange rate of about 0.5 times/h. Water was pretreated with gravel-packed filters and moving bed biofilm reactors in complement with UV radiation. To ensure that water parameters were consistently in optimal ranges, water quality (including NH_3_, ${\text{NH}}_{4}^{+}$, ${\text{NO}}_{2}^{-}$, ${\text{NO}}_{3}^{-}$) was monitored throughout the experiment, that is, temperature, 18.8–20.5°C; dissolved oxygen, 9.8–12.9 mg/l; and pH, 7.2–7.4. Whitefish were fed commercial dry pellets by automatic feeders distributing the food 12 h/day. We recorded no technical problems throughout the experiment.

For the comparison of Ab-staining patterns among salmonid fishes, we used rainbow trout [*Oncorhynchus mykiss* (Walbaum)] of the commercially available Troutlodge strain (http://www.troutlodge.com/; Tacoma, WA, USA). Trout were kept in 1,000-l tanks at 15°C in partially recirculating water systems and fed with commercial dry pellets.

### Sampling and Leukocyte Preparation

Fish averaging 21.6 ± 1.4 cm in length (mean ± STD) and 92.0 ± 24.7 g in weight were sampled at day 9 after the start of the experiments. Anesthesia and sampling corresponded to the standards of the German Animal Welfare Act [§ 4(3) TierSchG]. The entire liver, spleen and head and trunk kidney were isolated from seven animals from each group, sliced, and immediately frozen at −80°C until RNA isolation. Blood was collected from the caudal vein of four individuals per group using a heparinized syringe and immediately diluted in cold medium mixed with Iscove’s DMEM/Ham’s F12 (Gibco/Thermo Fisher Scientific, Darmstadt, Germany) at a ratio of 1:1. Head kidneys were homogenized to prepare single-cell suspensions. The cell suspensions were layered onto an isotonic Percoll gradient (Biochrom AG, Berlin, Germany) (*r* = 1.075 g/ml) and centrifuged at 1,800 rpm for 40 min. Cells at the Percoll/medium interphase were collected, washed with PBS–EDTA, resuspended in the corresponding volume of the medium or phosphate-buffered saline/ethylenediaminetetraacetic acid (PBS–EDTA) to the final concentration of 5 × 10^6^ cells/ml, and kept on ice until further preparation.

The spleno-somatic index (SSI) was calculated by the formula SSI = spleen weight (grams)/body weight (grams) × 100.

### Plasma Glucose Analysis

Blood samples were centrifuged (4°C, 1,700 rcf), and the supernatant was kept on ice until analysis of blood plasma parameters was performed. Plasma glucose concentrations were quantified using a colorimetric assay (Glucose Assay Kit II, BioVision, Milpitas, CA, USA) at the Beckman Coulter DTX 800/880 Series Multimode Detector (Beckman Coulter, Brea, CA, USA).

### Flow Cytometry and Immunomagnetic Leukocyte Sorting

For the analysis of the blood composition, 2 × 10^5^ cells/ml leukocytes were stained with a set of monoclonal antibodies (MAb) with known specificity for distinct subpopulations of rainbow trout leukocytes. The population of thrombocytes was stained by MAb 42 (36) and myeloid cells by MAb 30, and B cells were identified by MAb N2 (37) in complement with MAb 1.14 (38). Following the washing step, the cells were stained by secondary conjugates. Control aliquot was treated only with secondary conjugates. The samples were measured on BD FACSCanto II and analyzed using the DIVA software (BD Biosciences, Heidelberg, Germany).

Peripheral blood leukocytes obtained from five individuals were isolated as described above and subjected to immunomagnetic cell sorting according to the manufacturer’s protocol. Briefly, leukocytes were incubated in the presence of the monoclonal antibodies recognizing thrombocytes, myeloid cells, or B lymphocytes, followed by incubation with anti-IgG MicroBeads (Miltenyi Biotec, Bergisch Gladbach, Germany). Then, the cell suspension was applied to the MACS column, and the population of labeled cells was collected. The purity of enriched populations was estimated using BD FACSCanto II, and those exceeding 95% of labeled cells were used for the RNA isolation by the RNeasy Mini Kit (Qiagen GmbH, Hilden, Germany).

### Phagocytosis of the Latex Beads

To evaluate the phagocytic potential of the blood leukocytes, 100 µl blood was mixed with 5 µl latex beads (Sigma-Aldrich, Taufkirchen, Germany) labeled with fluorescein isothiocyanate (FITC). Blood cells were incubated for 2 h at 15°C in 2.5% CO_2_. Following the incubation, the cells were washed twice with PBS/EDTA. After the final washing, the cells were measured on BD FACSCanto II (BD Biosciences) and gated by forward and size scatter. Only FITC-positive cells were considered phagocytic cells, and their proportion was calculated relative to the total number of acquired leukocytes.

### Stimulation Experiments with *A. salmonicida*

The *Aeromonas salmonicida* ssp. *salmonicida* wild-type strain JF 2267 was used for stimulation trials. Bacteria were prepared according to the protocol described previously (39). *A. salmonicida* were either kept viable or inactivated in 1.5% paraformaldehyde (PFA) for 1 h. Prior to usage, the bacteria were diluted to a final concentration of 5 × 10^7^ cells/ml in sterile PBS.

Head kidney leukocytes from each individual were stimulated with 1 × 10^6^ viable or PFA-inactivated *A. salmonicida* ssp. *salmonicida*. An amount of 100 µl PBS was added to the control sample. After inoculation, the samples were incubated in a CO_2_ incubator at 15°C. The stimulated samples were collected after 12 h and stored in a 700 µl RLT buffer until RNA preparation.

### RNA Extraction

For RNA isolation, tissue samples were homogenized individually in 1 ml TRIzol Reagent (Invitrogen/Thermo Fisher Scientific) and purified using the RNeasy Mini Kit (Qiagen GmbH, Hilden, Germany) with 30 min on-column DNase treatment. The concentration and quality of RNA were proven using NanoDrop ND-1000 (NanoDrop Technologies/Thermo Fisher Scientific) and the Agilent 2100 Bioanalyzer (Agilent Technologies): only RNA samples with RIN values >9 were used for subsequent analyses. RNA was stored at −80°C.

### Microarray Experiments

Microarray experiments were conducted in duplicate; two biological replicates were performed for each tissue (liver or kidney) and stocking density (LD, MD, ED, or HD). To this end, seven individual RNA samples from the same tissue and SD were pooled. These RNA pools were individually used as template to produce Cy3-labeled cRNA according to the Low Input Quick Amp Labeling Kit (Agilent Technologies). Yields of the cRNA and the dye-incorporation rate were measured using the ND-1000 Spectrophotometer. The hybridization procedure was performed according to the One-Color Microarray-Based Gene Expression Analysis protocol (version 6.6, part number G4140-90040) using the Agilent Gene Expression Hybridization Kit (Agilent Technologies). In brief, 600 ng Cy3-labeled fragmented cRNA in a hybridization buffer was hybridized overnight (17 h, 65°C) to 8 × 60K Agilent-049158 Salmon Oligo Microarrays (Agilent Technologies; GEO platform: GPL21057) using Agilent’s recommended hybridization chamber and oven. After hybridization, the microarrays were washed once with the Agilent Gene Expression Wash Buffer 1 (room temperature, 1 min), followed by a second wash with the preheated Agilent Gene Expression Wash Buffer 2 (37°C, 1 min).

The fluorescence signals of the hybridized Agilent microarrays were detected using Agilent’s Microarray Scanner System G2505C (Agilent Technologies). The Agilent Feature Extraction software (version 10.7.3.1) quantified the intensity of the fluorescent images and normalized the results by subtracting the local background fluorescence based on a two-sided Student’s *t*-test. Data were then imported into a Rosetta Resolver gene expression data analysis system (Rosetta Biosoftware, Kirkland, WA, USA) for quality control and analysis.

The limma package of the R version 3.1.1/Bioconductor suite (40) was used to compare transcript abundances under MD and HD conditions. To control the false discovery rate, *p*-values were adjusted according to Benjamini and Hochberg (41). Genes were classified as differentially expressed if a corrected *p*-value threshold (*p* < 0.05) and an absolute fold change (FC > 1.5) met the criteria. Comparisons of gene expressions across different stocking densities were performed using Venn diagrams (42). Sets of differentially expressed genes were reannotated using the Basic Local Alignment Search Tool (BLAST). Only transcripts with unique BLAST results (coverage and sequence identity of >80% and E-value < 1 × 10^–4^) were included, and redundant probes representing identical transcripts were joined.

Gene lists were assigned to functional pathways using the Ingenuity program (Ingenuity Pathway Analyses, Ingenuity Systems/Qiagen), well minding that this program extracts global functional networks and canonical pathways of the differentially expressed genes according to investigations into mammalian, not teleostean, *in vivo* and *in vitro* systems. Enriched pathways were hence carefully reviewed and are indicated in the following by italic face; pathways of mammalian diseases were excluded from the analysis. The significance values were calculated using Fisher’s exact test right-tailed. Standard scores (*z*-scores) were used as a basis to assess whether certain pathways were activated (*z* > 1) or inhibited (*z* < 1); for pathways with *z*-scores around 0, no prediction could be made.

### Quantitative Real-time PCR

The concentration of total RNA was accurately determined in repeated measurements using the NanoDrop ND-1000 Spectrophotometer (NanoDrop Technologies) and the Agilent 2100 Bioanalyzer (Agilent Technologies). Subsequently, cDNA was synthesized from total RNA using the SuperScript II Reverse Transcriptase Kit (Invitrogen/Thermo Fisher Scientific) according to the supplier’s instructions.

Quantitative real-time PCR was performed using the LightCycler 480 Real-Time PCR System (Roche Diagnostics GmbH, Grenzach-Wyhlen, Germany) and SensiFAST SYBR No-ROX One-Step Kit (Bioline GmbH, Luckenwalde, Germany). The reaction mix contained 6 µl of 2 × SensiFAST SYBR No-ROX Mix (Bioline GmbH), 10 µM of each primer (see Table 1) and a cDNA equivalent of 75 ng total RNA (for the measurement of *CKM2, FDPS, GAMTb, IGF1, SAA5, STEAP4, TLR8a1*), 20 ng total RNA (*IL1B*), 10 ng total RNA (*CXCL8, TNF*), or 8 ng total RNA (*IRGA2B, CSF3R, IGM, TCR*). The temperature profile was as follows: initial denaturation step at 95°C for 10 min followed by 40 cycles with 15 s denaturation at 95°C, 10 s annealing at 60°C, and 20 s extension time at 72°C. The relative transcript amounts of the target genes were calculated and normalized against the reference gene *RPL9* (43) with the GeNorm stability value M = 0.43 and the coefficient of variation, CV = 0.15. The specificity of the primers was tested in a separate PCR experiment; the respective PCR products were sequenced, and the identity was verified using the BLAST algorithm. Quantitative PCR data were calculated using the qbase + software (Biogazelle, Ghent University, Belgium). The statistical significance was assessed using one-way analysis of variance (ANOVA) and is represented with “+” for *p* ≤ 0.05 and with “*” for *p* ≤ 0.01. The heat map displaying qRT data of *SAA5, IL1B, CXCL8*, and *TNF* was generated using the heatmap.2 function of the gplots R package.

**Table 1:** **Gene-specific quantitative real-time PCR primers used in this study**.

Gene symbol	GenBank accession #	Forward (5′–3′)	Reverse (5′–3′)	Amplicon length (bp)
**Validation of the array-predicted differential expression**				
*CKM2*	LN612738	CGTCTGCAGAAGCGTGGCAC	TGCTGTCGATGGCCTCTCCC	181
*FDPS*	Unpublished	TAGTTTGGACTGTGAGTGATCCT	GATGACATGATATTCCCAGTTTGA	156
*GAMTb*	LN612737	CTGAAGCCCAGTGGCGTTCT	GGTTGTGGTGCTAATCATCTCC	156
*IGF1*	LN812808	TATTGTGGACGAGTGCTGCTTC	CTCTGTCGACGCTTTGCACTG	163
*SAA5*	LN624222	TTCCCTGGTGAAGCTGCTCGA	TGACTCCTGCTGCCCACCTG	157
*STEAP4*	LK054751	GGCTTCCTTCCTGCAGCTCTA	TCAGTGACCCAGAACATCAGATA	177
*TLR8a1*	LN610596	ACCGGCTTTGAAAGTACTGA	CGTCCCTCTCTTTCCATAATGTG	134
**Validation of the identity of leukocyte populations**				
*IRGA2B*	Unpublished	GACAAGCAAGCATAAACAACTATC	TGCAGTTATAGGGAGTAAAACAAG	157
*CSF3R*	Unpublished	ACAGCCACTCCTGGAGGACG	GCCAAAGCCCTAAGCAAGGGA	177
*IGM*	Unpublished	AATTCCAATTCATTGAGCCAACTC	TCCCTCACGTTCGTCATATTCTT	158
*TCR*	Unpublished	GTAAAAGATGACATTGCAGGTGAA	CAACGATCACAACAGAACTGAAG	151
**Cytokine profiling in stimulated head kidney leukocytes**				
*IL1B*	LN624221	AAGGACAAGGACCTGCTCAACT	ACCCAGCTCTTGTTCTCAGAGT	160
*CXCL8*	LN624218	CTGAGGGGATGAGTCTGAGAG	ATCTCCTGACCGCTCTTGCTC	168
*TNF*	Unpublished	GATACCCACCATACATTGAAGCA	ATTTGGTTCCCCTGTAGCTCGA	162
**Reference gene**				
*RPL9*	HE984307	ACCACATCAACCTGGAACTCA	CGCATCTTGTAACGGAAACC	162

## Results

### Crowding Stress Induces Immune Pathways in the Liver and Kidney of Whitefish

The microarray technology was used to record the global expression changes induced by different SDs in the liver and kidney of maraena whitefish. Only annotated genes (between 85 and 100% of all regulated features) with an absolute FC > 1.5 and corrected *p*-values < 0.05 were considered.

**Figure 1:**
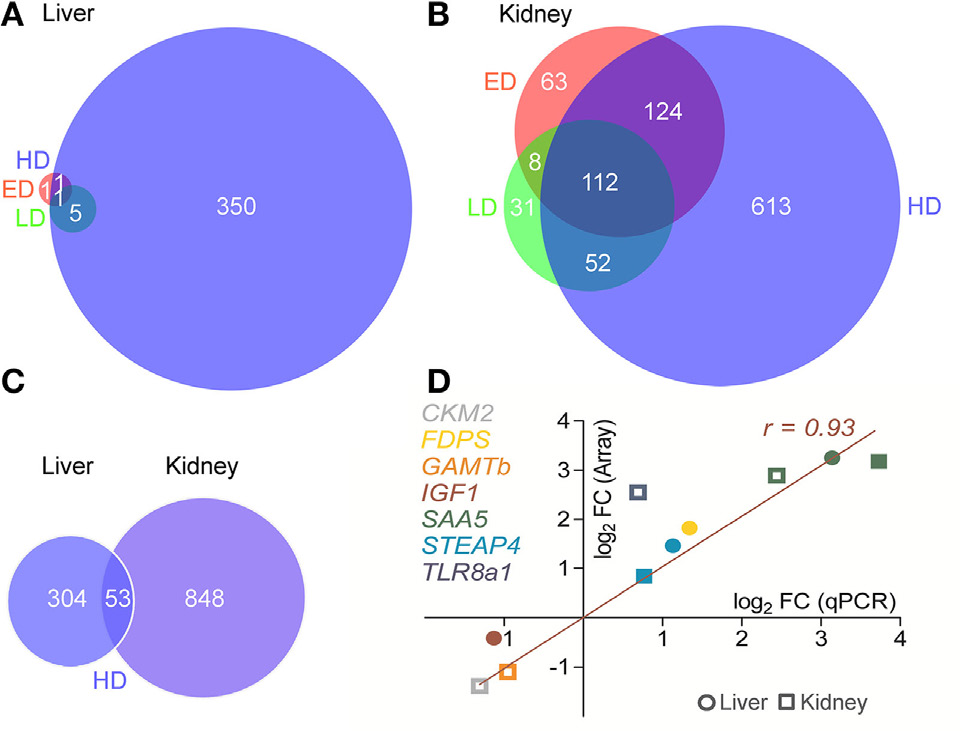
**Venn diagrams show the overlap in differentially expressed genes at different stocking densities (SDs) in the (A) liver and (B) kidney of maraena whitefish kept at low density (green), elevated density (ED) (red), and high density (HD) (blue) each compared with moderate density (MD) conditions**. The intersections indicate the number of genes affected by more than one SD. Criteria for significance were a total fold change (FC) > 1.5 and a corrected *p* < 0.05 of annotated, non-redundant features. **(C)** In a subsequent analysis, the sets of differentially expressed genes of HD vs. MD treatment were compared between liver and kidney. **(D)** The concordance of array (ordinate) and quantitative real-time PCR (abscissa) FC values for selected genes (as listed on the right) were plotted for ED vs. MD (open symbols) and HD vs. MD (filled) comparisons in the liver (circular) or kidney (squared). Pearson’s correlation is indicated.

In the liver, 357 genes were affected by HD conditions (168 up- and 189 downregulated genes relative to MD), while only 6 and 3 genes were differentially expressed under ED and LD conditions, respectively (Figure 1A). As opposed to that, the number of differentially expressed genes in the kidney was clearly higher under HD (396 up- and 507 downregulated genes), ED (102 up- and 207 downregulated genes), and LD (11 up- and 192 downregulated genes) conditions compared with MD (Figure 1B). The liver and kidney shared 53 differentially expressed genes in the HD group, while sets of differentially expressed genes neither from LD nor from ED groups showed overlaps across the two selected tissues (Figure 1C).

The full complement of microarray data is available in the Gene Expression Omnibus database (GEO accession: GSE76543). Quantitative RT-PCR was used to validate the array-predicted expression differences of a select panel of whitefish genes publicly available at GenBank, that is, *CKM*, variant 2; *FDPS*; *GAMT*, variant b; *IGF1*; *SAA5*; *STEAP4*; and *TLR8*, variant a1 (Table 1; Figure 1D). Student’s *t*-test validated a significantly different expression (*p* < 0.05) for all selected genes, except for *IGF1* (*p* = 0.07). Accordingly, profiles across all target genes revealed a high concordance (Pearson product-moment correlation coefficient, *r* = 0.93; coefficient of determination, *r*^2^ = 0.87).

Assigning differentially expressed genes to functional groups revealed that numerous stress-related signaling pathways were activated in the liver and kidney under ED and HD conditions (Table 2), such as *ERK/MAPK, mTOR, glucocorticoid receptor, SAPK/JNK*, and *JAK/Stat signaling*, as well as *p38* and *p53 signaling*. Moreover, several stress-relevant effector pathways were found to be overexpressed, including *glycolysis, gluconeogenesis, glycogen degradation*, and *ascorbate recycling*. The induction of a glycolytic pathway is, however, not supported by the measured plasma glucose levels, showing no significant differences in the four sets of fish exposed to the different SD conditions (data not shown).

**Table 2:** **Potential stocking density (SD)-induced Ingenuity pathways (*p* < 5.0E−2) regulated in whitefish held at ED or HD compared with MD conditions**.

Canonical pathway	*p*-Value	*z*-Score	SD^a^	Involved DE genes^b^
**Overexpressed in liver**				
ERK/MAPK signaling	1.5E−4	0.91	HD	11 (187)
p53 signaling	4.0E−3	0	HD	6 (98)
JAK/Stat signaling	2.5E−2	0	HD	4 (72)
p38 MAP kinase signaling	3.4E−2	0.45	HD	5 (117)
**Overexpressed in kidney**				
Glycolysis	2.2E−4	0	ED	11 (25)
Glycolysis	3.8E−10	0	HD	4 (25)
Glucocorticoid receptor signaling	5.5E−4	0	ED	11 (275)
Glucocorticoid receptor signaling	3.1E−6	0	HD	28 (275)
Gluconeogenesis	3.3E−3	0	ED	3 (25)
Gluconeogenesis	7.6E−9	0	HD	10 (25)
Glycogen degradation	1.4E−5	0	HD	5 (12)
JAK/Stat signaling	2.7E−4	1.27	HD	10 (72)
Stress-activated protein kinase/JNK signaling	6.1E−4	1.27	HD	11 (94)
p38 MAP kinase signaling	3.6E−3	1.00	HD	10 (117)
Glutamate degradation	3.9E−3	0	HD	2 (3)
HIF1-alpha signaling	4.0E−3	0	HD	10 (102)
Ascorbate recycling	4.8E−2	0	ED	1 (4)

*^a^Treatment ED (elevated density) or HD (high density) compared with MD (moderate density)*.

*^b^1.5 > FC < −1.5; corrected p < 0.05; the total number of pathway-involved genes is given in brackets*.

A high number of 43 out of 168 upregulated genes in the liver (~26%) and 80 out of 396 upregulated genes in the kidney (~20%) from fish kept at HD (compared with MD fish) were related to immunological processes. Particularly worth mentioning here is the strong upregulation of a chemotaxin-encoding gene (*LECT2*) in the liver (23.6-fold), an acute-phase gene encoding serum amyloid protein A-5 (*SAA5*) in the in liver (8.8-fold) and kidney (13.3-fold), a lysozyme-encoding gene (*LYZ*) the liver (3.5-fold) and kidney (8.6-fold), the complement factor-encoding genes *C7* (7.6-fold) and *C1Q*-like (4.0-fold) in the liver, a cytokine-encoding gene (*CCL19*) in the liver (4.9-fold), and a transcription factor-encoding gene (*CEBPB*) in the liver (2.4-fold) and kidney (3.9-fold). On the other hand, genes encoding antiviral effectors such as the myxovirus resistance factor (MX1) and the influenza virus-binding protein IVNS1ABP were about two-fold downregulated in the kidney. The IPA program was again used to infer which pathways may have been influenced by the regulation of the immune genes regulated in the expression (setting a cutoff *p*-value < 1 × 10^−4^). Eight immune pathways were identified as activated at HD in the liver; 4 and 13 immune pathways were activated at ED and HD in the kidney, respectively (Table 3). Correlating with the comparatively low number of downregulated immune genes, only a few immune pathways were inhibited, such as *CD40 signaling* in the liver of fish kept at HD (Table 4).

**Table 3:** **Activated immune-relevant Ingenuity pathways (with *p* < 1.0E−4) induced in whitefish held at ED or HD compared with MD conditions**.

Canonical pathway	*p*-Value	*z*-Score	Stocking density^a^	Involved DE genes^b^
**Overexpressed in liver**				
Acute-phase response signaling	1.3E−10	2.67	HD	18 (169)
Protein kinase C-θ signaling in T lymphocytes	4.0E−6	0.30	HD	11 (118)
Interleukin-6 signaling	1.2E−5	1.27	HD	10 (116)
Complement system	1.9E−5	1.34	HD	7 (37)
Phosphoinositide 3-kinase signaling in B lymphocytes	2.8E−5	1.00	HD	10 (128)
iCOS-iCOSL signaling in T helper cells	4.2E−5	0.82	HD	9 (108)
B cell receptor signaling	8.0E−5	0.30	HD	11 (174)
CD28 signaling in T helper cells	8.6E−5	0.82	HD	9 (118)
**Overexpressed in kidney**				
Production of nitric oxide and reactive oxygen species in macrophages	1.3E−5	0.91	ED	11 (180)
Production of nitric oxide and reactive oxygen species in macrophages	9.3E−9	1.63	HD	25 (180)
Phagocytosis in macrophages and monocytes	1.7E−5	0.71	ED	8 (93)
Phagocytosis in macrophages and monocytes	5.7E−9	1.41	HD	18 (93)
*N*-formyl–methionyl–leucyl–phenylalanine (fMLP) signaling in neutrophils	5.1E−5	1.41	ED	8 (108)
fMLP signaling in neutrophils	1.9E−9	2.98	HD	20 (108)
Interleukin-8 signaling	8.5E−5	1.27	ED	10 (184)
Interleukin-8 signaling	1.5E−8	1.88	HD	25 (184)
CD28 signaling in T helper cells	5.1E−8	1.89	HD	19 (118)
Leukocyte extravasation signaling	6.4E−8	1.46	HD	25 (198)
Tec kinase signaling	2.7E−7	1.41	HD	21 (157)
High-mobility group protein B1 signaling	7.3E−6	1.81	HD	15 (120)
B cell receptor signaling	2.0E−5	0.94	HD	19 (174)
Acute-phase response signaling	4.6E−5	0.50	HD	17 (169)
Chemokine signaling	4.8E−5	0.91	HD	11 (71)
Role of pattern recognition receptors in recognition of bacteria and viruses	5.5E−5	1.94	HD	14 (126)
Interleukin-6 signaling	8.3E−5	1.60	HD	13 (116)

*^a^Treatment ED (elevated density) or HD (high density) compared with MD (moderate density)*.

*^b^1.5 > FC < −1.5; corrected p < 0.05; the total number of pathway-involved genes is given in brackets*.

**Table 4:** **Silenced immune-relevant Ingenuity pathways (with *p* < 5.0E−2) repressed in whitefish held at ED or HD compared with MD conditions**.

Canonical pathway	*p*-Value	*z*-Score	Stocking density^a^	Involved DE genes^b^
**Underexpressed in liver**				
CD40 signaling	5.2E−8	−1.41	HD	10 (65)
NF-κB signaling	3.5E−4	−1.67	HD	10 (173)
Activation of IRF by cytosolic pattern recognition receptors	2.8E−3	−0.45	HD	5 (63)
Tec kinase signaling	3.0E−3	−0.38	HD	8 (157)
Interleukin-17A signaling	3.0E−3	−0.45	HD	5 (64)
OX40 signaling pathway	1.2E−2	−0.45	HD	5 (89)
**Underexpressed in kidney**				
Integrin signaling	1.8E−4	−0.63	ED	10 (201)
Acute-phase response signaling	1.7E−2	−1.00	ED	6 (169)
Dendritic cell maturation	2.6E−4	−0.50	HD	17 (177)
Interleukin-22 signaling	1.5E−3	−1.34	HD	5 (24)

*^a^Treatment ED (elevated density) or HD (high density) compared with MD (moderate density)*.

*^b^1.5 > FC < −1.5; corrected p < 0.05; the total number of pathway-involved genes is given in brackets*.

**Figure 2:**
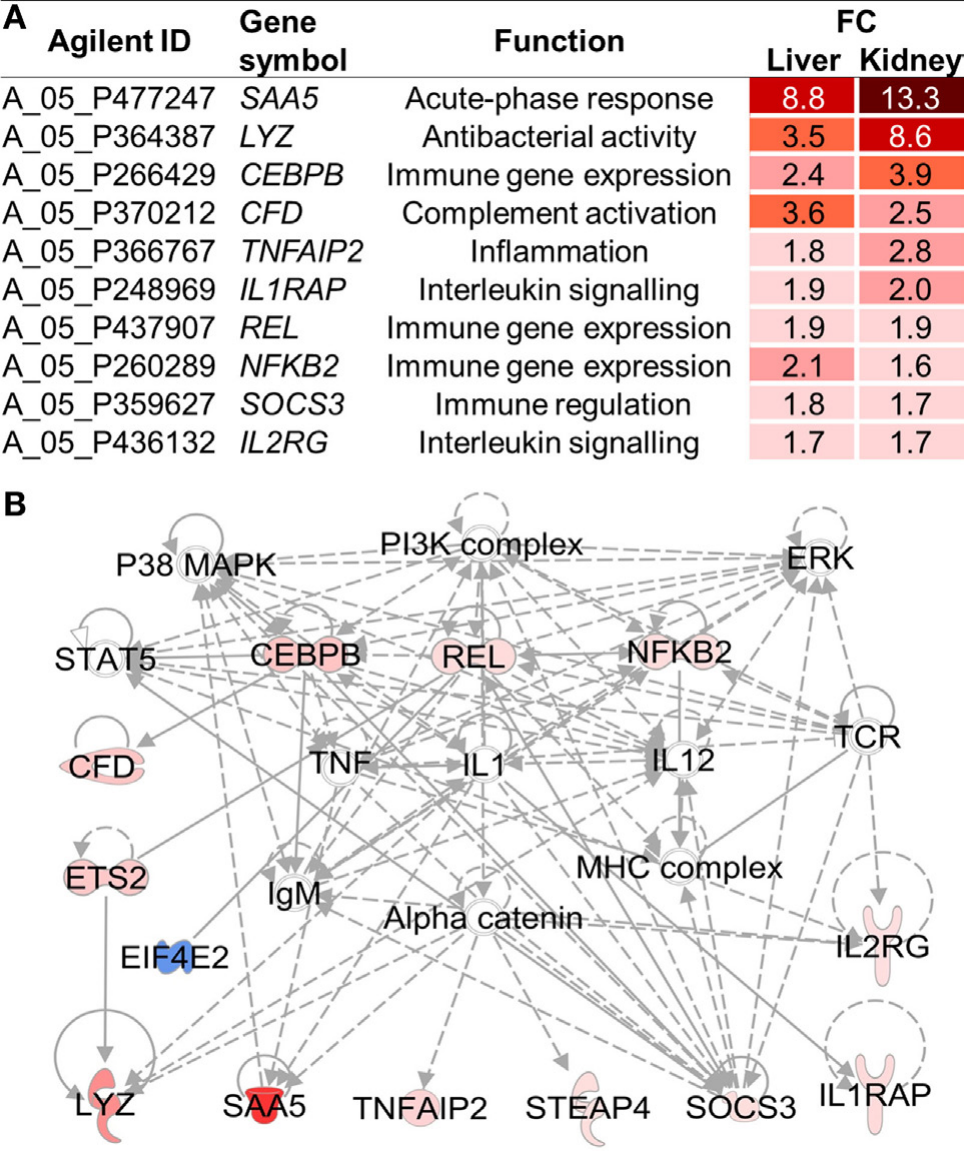
**(A)** Among the 53 high density (HD)-induced genes shared by the liver and kidney (*cf*. Figure 1C), 10 genes were involved in innate immunity, as assessed using the Ingenuity Pathway Analysis program. **(B)** Based on these 53 shared genes, an IPA network was constructed, including all 10 immune genes. Red and blue symbols indicate up- and downregulated genes, respectively, in HD vs. moderate density whitefish. Full and broken lines indicate direct and indirect relationships, respectively. Open and filled arrows represent an influence on translocation and activation as well as protein–protein or protein–DNA interactions, respectively.

Four of the activated pathways, *acute-phase response signaling, B cell receptor signaling, CD28 signaling in T helper cells*, and *interleukin-6 signaling*, were shared by the liver and kidney. Accordingly, 10 immune genes were commonly expressed in both tissues of fish exposed to HD (Figure 2A). A network analysis suggested that the expression of these shared immune genes is closely interrelated with the activation of stress-related kinases, above all p38 (Figure 2B). A central role has been assigned to interleukin-1 (IL1) as an extensively networked factor.

### Immune Cell Composition Is Altered in Stressed Whitefish

**Figure 3:**
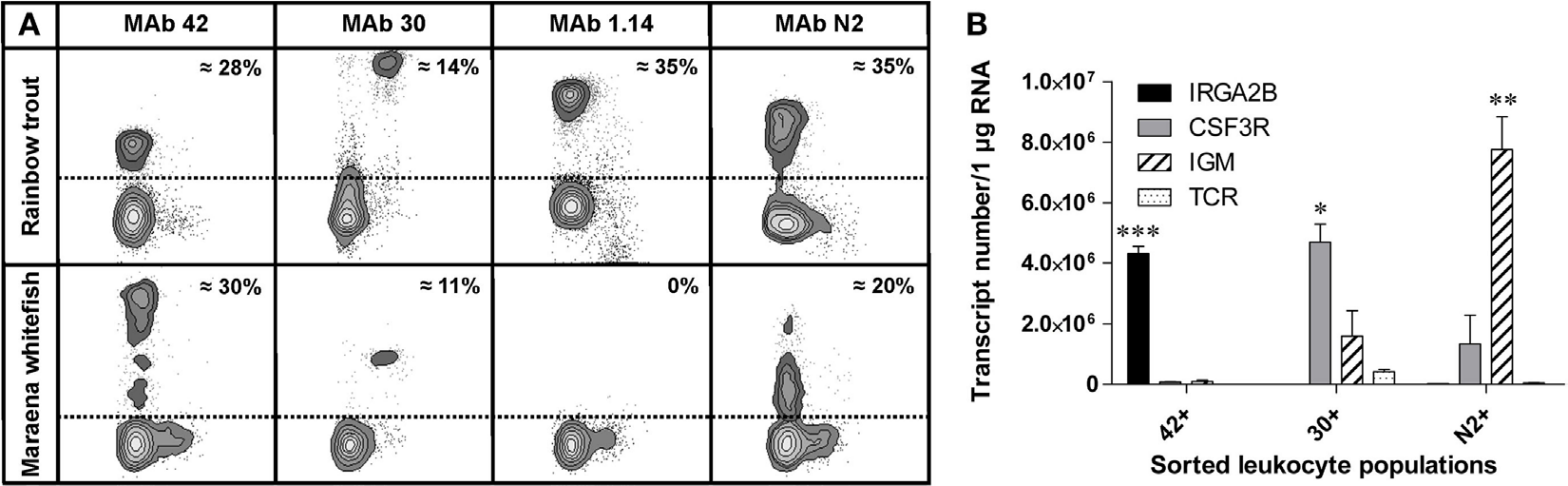
**(A)** Comparison of the staining patterns of the cross-reactive monoclonal antibodies detecting peripheral blood leukocytes of rainbow trout (upper panel) and maraena whitefish (lower panel). PBLs were stained with monoclonal antibodies MAb 42 (thrombocytes), MAb 30 (myeloid cells), MAb 1.14 (B cells; heavy chain of immunoglobulin), and MAb N2 (B cells; light chain). The proportion of each cell population is expressed as the percentage of the number of stained PBLs. **(B)** The copy number of the leukocyte marker genes *IRGA2B*/*CD41* (black bars), *CSG3R*/*GCSFR* (gray), *IGM* (hatched), and *TCR* (dotted) in immune cell populations magnetically sorted with MAb 42, MAb 30, and MAb N2 as indicated. Graphs depict the mean values + SEM (error bars). Transcript numbers significantly different from the other markers are indicated with asterisks, as evaluated by one-way analysis of variance (*p = 0.03; **p = 0.01; ***p = 0.0001).

The composition of blood leukocytes is considered a reliable marker for assessing the immune status during pathophysiological conditions in numerous species across the animal kingdom (44–46). Based on the high number of overexpressed immune pathways as a consequence of increased SD, we hypothesized that adverse husbandry conditions will affect the composition of leukocyte subsets. To prove this hypothesis, we first validated the suitability of the available immunological tools: an established panel of monoclonal antibodies recognizing leukocyte subpopulations in rainbow trout was tested for cross-reactivity with the leukocytes of maraena whitefish. These MAbs included the thrombocyte-specific MAb 42, myeloid cell-specific MAb 30, and MAb 1.14 and MAb N2 recognizing a heavy and a light chain of immunoglobulin IgM, respectively. These antibodies stained leukocyte subpopulations of whitefish similar to those of trout, except for MAb 1.14 (Figure 3). Moreover, the proportion of the cells recognized by each antibody was comparable between the two species: MAb 42 stained approximately 30% of the blood leukocytes (presumably thrombocytes); MAb 30 stained 11–14% of the leukocytes (myeloid cells); MAb N2 stained around 35% in trout and 20% in whitefish (B cells).

The identity of the magnetically sorted leukocyte populations was assessed *via* transcription profiling of marker genes encoding *IGM* in B cells, *CSF3R* (alias *GCSFR*) in myeloid cells, and *IRGA2B* (alias *CD41*) in thrombocytes. As expected, the highest level of transcripts encoding *IRGA2B* was detected in the population of MAb 42^+^ cells (thrombocytes), the large granular MAb 30^+^ leukocytes (myeloid cells) expressed the highest levels of *CSF3R*-encoding mRNA, and the highest transcript level of *IGM* was found in the population of MAb N2^+^ cells (B cells) (Figure 3B). Notably, low levels of *IGM* were detected in the population of large granulated leukocytes.

**Figure 4:**
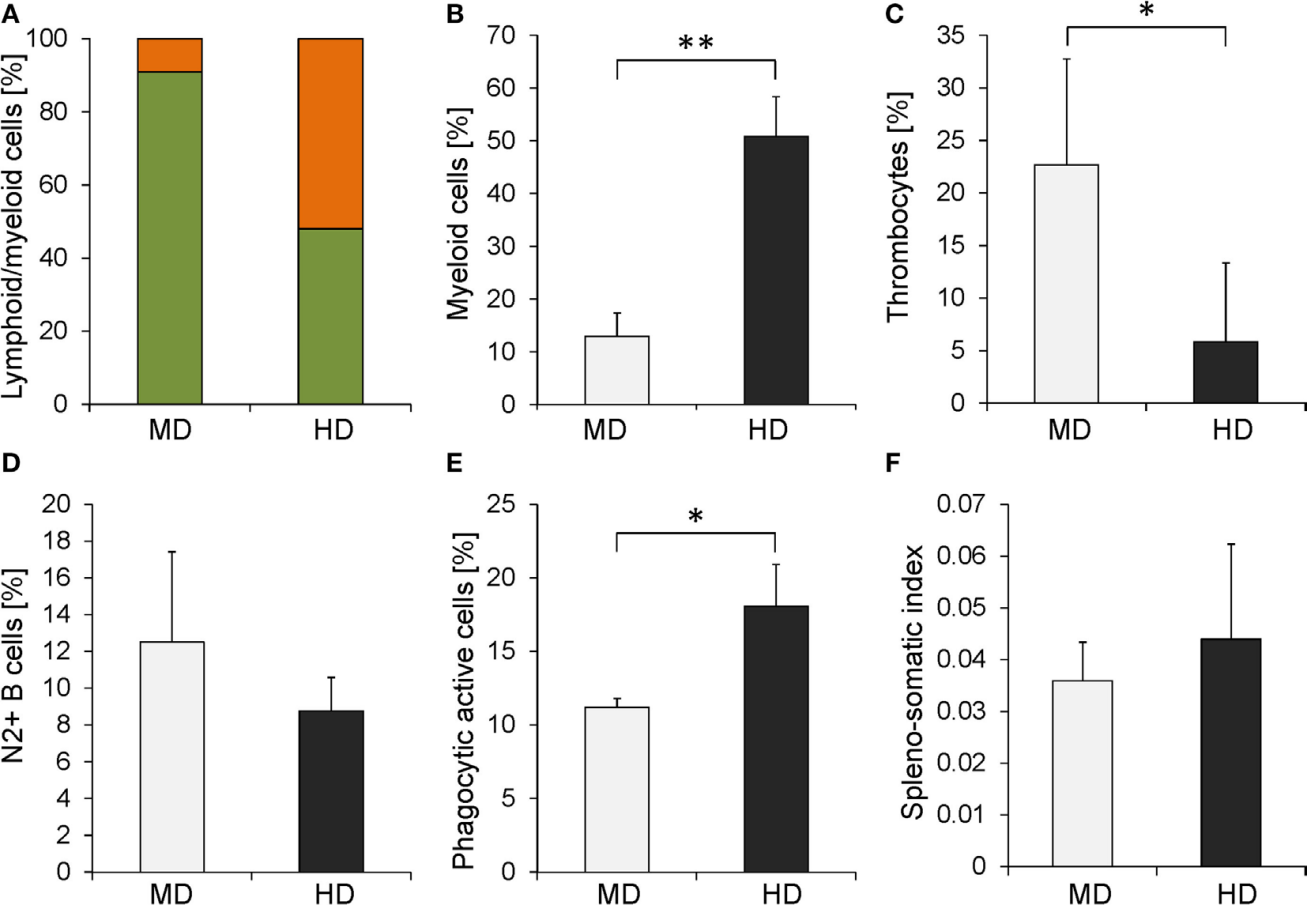
**Comparison of several immunological parameters between whitefish kept at moderate density (MD) (gray bars) and that kept at high density (HD) (black bars)**. **(A)** The ratio between lymphocytes (green filling) and myeloid cells (orange filling) was determined based on size and granularity using flow cytometry. Specific MAbs allowed the quantification of the proportion of **(B)** myeloid cells, **(C)** thrombocytes, and **(D)** light-chain-positive B cells. **(E)** The phagocytic activity of blood leukocytes was assessed using latex bead-based assays. **(F)** SSI was determined for MD and HD individuals. Graphs represent the mean values + stocking density (error bars). Significant changes calculated using one-way analysis of variance are marked with (*) for *p* ≤ 0.05 and (**) for *p* ≤ 0.01.

Given that functional set of cross-reactive antibodies, we evaluated the impact of crowding on the composition of the peripheral blood. To this end, blood was sampled from five fish per HD or MD group and analyzed using flow cytometry. The acquired cells were plotted in the forward and the side scatter to estimate the proportion between small cells with low granularity, presumably lymphocytes, and larger granulated cells of myeloid origin. This approach revealed that the PBLs of MD individuals comprise more than 91% lymphocytes, while myeloid cells represent approximately 9% of all gated leukocytes (Figure 4A). This ratio shifted considerably in HD individuals: myeloid cells represented with 52% a majority of all gated PBLs, while the lymphocytes represented 48%. Subsequently, we analyzed the blood composition in greater detail using the MAb repertoire described above. The highest difference between MD and HD groups was detected by the myeloid cell-specific MAb 30 (Figure 4B). Fish kept at HD showed a higher proportion of myeloid cells (50% of all gated cells) compared with fish kept at MD (13%). The proportion of thrombocytes was higher in MD fish (22%) compared with that in HD fish (5%) (Figure 4C), and the proportion of B cells was slightly higher in the MD group (12%) compared with that in the HD group (9%) (Figure 4D).

### Crowding Stress Elevates the Phagocytic Activity of Whitefish Leukocytes

Among the IPA-predicted pathways in the kidney of maraena whitefish exposed to increased SD was the *phagocytic activity in macrophages and monocytes* (*z* = 1.41; Table 3), as well as *actin nucleation by ARP-WASP complex* (*z* = 0.83), *regulation of actin-based motility by Rho* (*z* = 0.78), and *phagosome formation* (*z* = 0), indicating enhanced cell locomotion combined with the internalization of foreign material. Isolated PBLs were thus subjected to a phagocytic assay with fluorescent-labeled latex beads mimicking host–pathogen interaction during infectious diseases to study the leukocytes’ potential to internalize infectious agents and trigger proinflammatory processes (Figure 4E). The percentage of cells taking up the particular antigen was lowest in the MD group (11%), while it significantly increased in the HD group (18%).

### Spleens of Stressed Whitefish Are Not Significantly Enlarged

The induced expression of angiotensinogen (*AGT*, 4.5-fold) in the kidney and the overall dramatic changes in the expression of surface molecules involved in cell adhesion and migration (*CD9, CD81, CD63, CD166*) together with the elevated level of myeloid cells in HD individuals may indicate accompanying cell migration into the SP as a main secondary lymphoid organ of salmonid fish. We investigated, thus, whether fish from MD and HD groups exhibited a difference in SP size. Although we found a slightly increased spleno-somatic index (SSI) in the HD group, the difference was not statistically significant (Figure 4F).

### Head Kidney Leukocytes Show Distinct Expression Profile of Proinflammatory Cytokines Reflecting Post-Stress Conditions

**Figure 5:**
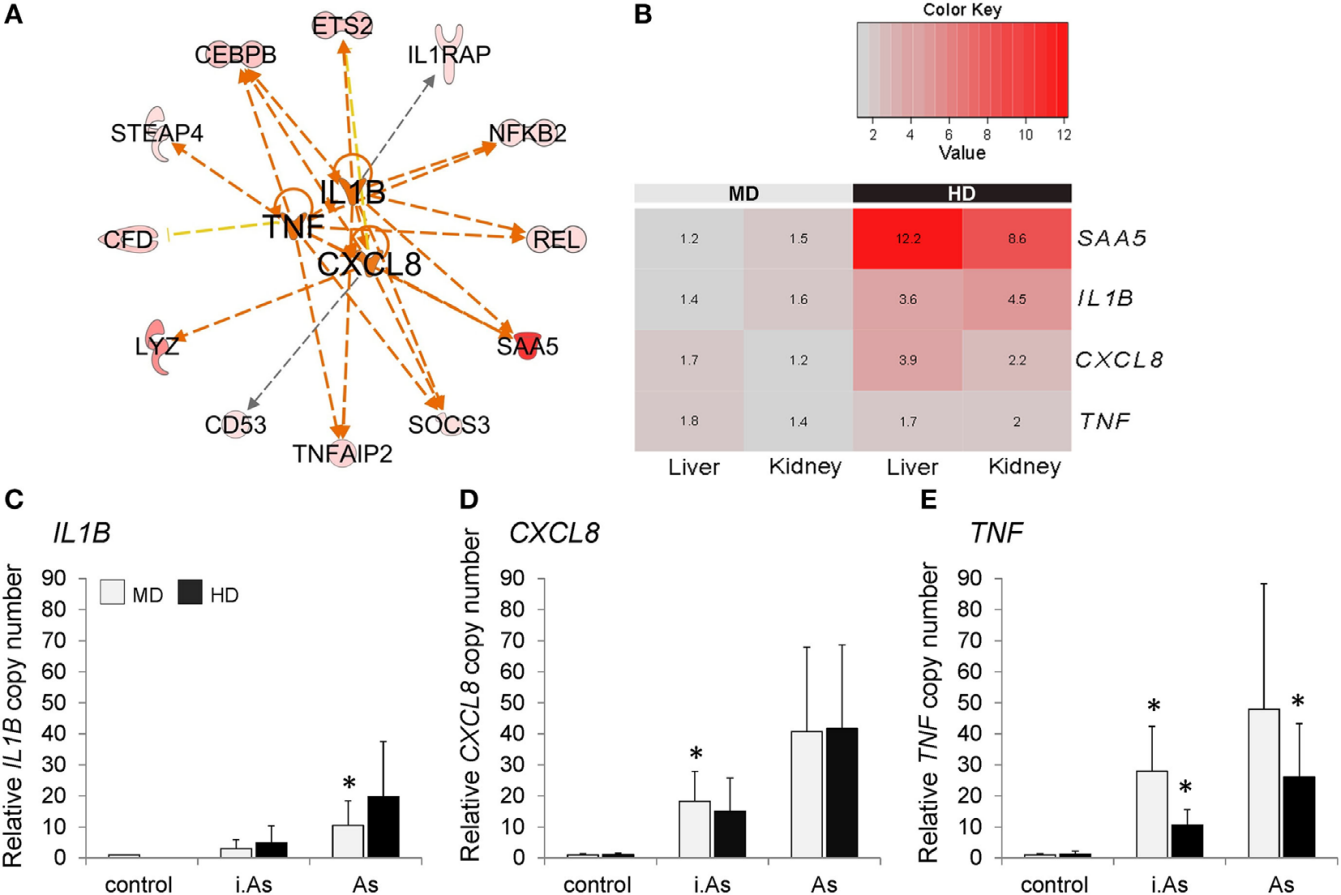
**(A)** IPA upstream regulator analysis predicted *IL1B, CXCL8*, and *TNF* to control the overexpression of 12 genes in both liver and kidney in high density (HD) conditions compared with those in moderate density (MD) conditions. Orange lines indicate an activating impact on gene expression, while yellow and black lines indicate inconsistent or non-predicted effects. The network was manually edited; please note that ingenuity analysis is based on literature about mammalian molecules. **(B)** The heat map displays expression ratios [given as fold change (FC)] of *SAA5, IL1B, CXCL8*, and *TNF* (normalized to RPL9) in the liver and head kidney of maraena whitefish (*n* = 8). The bar above the heat map indicates the density categories analyzed. Scaled color intensity within each row verifies high FC > 2.0 (shades of red). Significance levels were determined using analysis of variance (ANOVA) analysis and Bonferroni posttest. The transcription levels of **(C)** *IL1B*, **(D)** *CXCL8*, and **(E)** *TNF* were evaluated in head kidney leukocytes 12 h after cocultivation with inactivated (i.As) and viable *A. salmonicida* (As). Leukocytes had been isolated from fish kept at MD (light gray bars) or HD (black). Graphs depict the mean values + SD (error bars). Significant changes (*p* ≤ 0.05) compared with controls are marked with “*” as evaluated using one-way ANOVA.

The above evidence suggests a “priming” of the innate immune system in whitefish on crowding stress. An *in vitro* approach was used to evaluate whether leukocytes from presumably stressed whitefish develop a stronger inflammatory response than leukocytes from unstressed animals. To this end, head kidney leukocytes were isolated from whitefish previously kept either at MD or at HD. These cells were subsequently challenged with live or inactivated *A. salmonicida*. The induction of inflammation was validated, measuring the expression of genes encoding the proinflammatory cytokines IL1 beta (*IL1B*), interleukin-8 (*CXCL8*), and tumor necrosis factor alpha (*TNF*). These cytokines have been predicted to control the expression of 12 genes, which have been identified as differentially regulated under MD compared with HD conditions (Figure 5A). Moreover, we found that the mRNA levels of these proinflammatory cytokines tended to be upregulated in HD compared with MD fish, though non-significantly (Figure 5B).

The mRNA levels of the genes investigated were not different in head kidney leukocytes from both MD and HD groups prior to stimulation. Twelve hours poststimulation, the transcript levels of *IL1B, CXCL8*, and *TNF* were increased in leukocytes from both groups. Here, it became clear that the infection with inactivated *A. salmonicida* induced generally lower transcription levels than stimulation with viable bacteria (Figures 5C–E). Following the challenge with viable bacteria, *TNF* and *CXCL8* were upregulated with fold-change values above 40, while *IL1B* was only (but still) 10.5-fold induced. Remarkably, *TNF* transcripts reached after stimulation (with viable and inactivated bacteria, respectively) a higher level in the MD group, which was approximately twice as high as measured in the HD group (Figure 5E). A similar effect was not reflected by the *IL1B* and *CXCL8* expression profiles (Figures 5C,D).

## Discussion

Higher stocking densities in intensive aqua farming promise higher yields, but there are several reports citing complications of the production process involving immune suppression in farmed fish, which in turn trigger the outbreak of diseases (47, 48). However, sensitivity to stress differs among species and between populations depending on their evolutionary history (30). Maraena whitefish *C. maraena* represents a relatively young aquaculture species. We elucidated the physiological effect of a 9-day exposure to SDs between 10 and 100 kg whitefish per cubic meter with the primary goal to identify a comprehensive set of indicators characterizing the health status of farmed maraena whitefish with respect to adverse husbandry conditions. This study thus provides novel insights addressing the overarching question of whether the crowding stress-related regulation of the immune response is conserved throughout the evolution of vertebrates.

Global transcriptome profiling revealed that the number of differentially expressed genes affected by crowding was higher in the kidney of whitefish than in the liver; moreover, the proportion of downregulated genes was slightly higher in the liver (113%) and kidney (128%) compared with the number of upregulated genes. Although stress conditions had a negative impact on a number of pathways, crowding activated several signal transduction pathways, including signaling through the stress-activated protein kinase or the “stress kinase” p38, which are all well-known to be switched on by cellular stress [reviewed in Ref. (49–53)]. Concomitantly, we detected a significant impact on the immune system. In both the liver and the kidney tissue of whitefish exposed to HD, a strikingly high number of differentially expressed genes were related to immunity, mainly *acute-phase response, TNF* and *interleukin signaling*, and *complement pathways*. Regarding the latter, it may be noteworthy that the involved regulated features belong to the classical (*C1Q, C4B*) and the alternative complement pathway (*CFB, CFD*), which are both also present in salmonid fish (54). In this regard, it should be stressed that only 53 genes were commonly regulated in both organs analyzed, suggesting that the responses to increased SD are tissue-specifically organized.

Previous holistic studies on the transcriptomic responses in teleost liver and kidney revealed clear stress-immune interactions [reviewed in Ref. (15)]. *Acute-phase* and *interleukin signaling* as well as *complement pathways* are among the most frequently observed immune pathways overrepresented upon stress exposure in salmonid fish (55–57). The interdigitation of stress- and immune-related pathways is a well-documented phenomenon across different animal models and microarray platforms, indicating a generally conserved response of the immune system to overcome stressful conditions. In this respect, a key role is attributed to the sympathetic nervous system, which delivers stress-responsive neurotransmitters to hematopoietic and lymphoid tissues, thereby, in essence, stimulating the expression of proinflammatory genes but suppressing antiviral responses (26). Recent studies in mammals demonstrated that the proliferation of myeloid cells (mainly monocytes and granulocytes) is upregulated in the bone marrow through β-adrenergic signaling and thus structures a characteristic stress response pattern (26–29, 58, 59). The present study points to a similar response pattern of stress-induced myelopoiesis in maraena whitefish, proving a comparably strong increment in myeloid cell number in individuals exposed to high densities. As a prominent feature, we suggest, in addition, the increased phagocytic activity of those leukocytes from stressed whitefish. In this respect, reference should be made to *LECT2*, the most strongly upregulated gene in the liver of whitefish kept under HD conditions. The increased expression of *LECT2* in the liver of stressed trout has been documented earlier (60), and it has been demonstrated in mouse *in vitro* models that LECT2 treatment enhanced the phagocytosis and bacterial killing of macrophages (61). Increased phagocytic function could be analogous to the change in myeloid cell differentiation and effector function previously observed in socially stressed mice (28, 62).

Stress-related alterations of the PBL composition are an evolutionarily conserved feature. The mobilization of myeloid cells and their release into the bloodstream has also previously been observed in gilthead seabream (*Sparus aurata*) after exposure to short-term stress (23). In stressed rats, it has been demonstrated that the proportion of myeloid cells is influenced by decreasing the number of lymphocytes and monocytes together with the increasing number of neutrophils (63). These effects are enhanced by stress hormones also altering hematopoiesis and leading to the apoptosis of T and B lymphocytes as well as the expansion of myeloid cell number (64). Since no reliable markers for the distinction between monocytes, macrophages, neutrophils, and their precursors are available yet for fish, the production of the respective Abs should be addressed in the future to gain deeper insights into cellular upheavals during all kinds of imbalanced physical conditions.

Our data indicate not only an increased number of myeloid cells in whitefish exposed to crowding stress but also expression profiles equating to the CTRA (26, 27), including (i) enhanced proinflammatory activities (activated pathways *interleukin-8 signaling, acute-phase response signaling, chemokine signaling*), (ii) increased T-lymphocyte activation (activated *Tec kinase* and *CD28 signaling*), and (iii) an increased activity of NF-κB transcription factors (upregulated genes coding for NF-κB p50 and −p65), concomitant with (vi) a reduced antiviral response (downregulated genes encoding myxovirus resistance factor MX1 and influenza virus–binding protein IVNS1ABP) as evaluated for the hematopoietic, immune-, and stress-relevant kidney of whitefish exposed to crowding. These observations are essentially in accordance with the abovementioned reports about stress in mammals (*cf*. Table 5), suggesting that the mechanisms by which stress regulates immune responses may have been established earlier in the evolution (27, 28, 58). It is also worth noting that we have seen enhanced antimicrobial activities in the kidney of density-stressed whitefish (activated pathways’ *complement system* and *production of NO and ROS in macrophages*), which is broadly consistent with both the increased proinflammatory signaling noted above and previous measures of antimicrobial response in socially stressed mice (62). However, in contrast to results from mammalian systems (28, 65), we did not find any indications of a reduced *glucocorticoid-mediated signaling*, which has been observed in stressed mammals (28, 65). In fact, IPA analyses indicated increased glucocorticoid-related signaling in the kidney for crowded fish. Nonetheless, the activation of the stress-induced proinflammatory pathways is related to substantial energetic costs presumably covered by those energy-mobilizing actions (*glycolysis, gluconeogenesis, glycogen degradation*) recorded in stressed whitefish in the present study and generally characterizing fish under stress conditions (20, 66–68).

**Table 5:** **Comparison of conserved transcriptional response to adversity (CTRA) characteristics in stressed whitefish and stressed mammals (compared with unstressed controls)**.

CTRA patterns	Whitefish (present study)	Mammals	Reference
Transcriptional changes	Liver and kidney: 1.1- to 1.3-fold more downregulated than upregulated features	Murine spleen monocytes: 1.5-fold more downregulated than upregulated features	Powell et al. (28)
Activity of transcription factors mediating inflammatory functions	Increased	Increased in mice Increased in rhesus macaque	Powell et al. (28), Cole et al. (58)
Expression of proinflammatory genes	Increased	Increased in mice	Powell et al. (28)
Expression of antiviral genes	Decreased	Decreased in mice Decreased in rhesus macaques	Powell et al. (28), Cole et al. (58, 59)
Proportion of myeloid cells in circulation	Increased	Increased in humans Increased in rats Increased in mice	Heidt et al. (29), Engler et al. (65), Powell et al. (28)
Rate of phagocytosis	Increased	Increased in rats Decreased in mice	Stefanski and Grüner (69), Bailey et al. (62)
Reaction after bacterial challenge	Higher expression of *IL1B*, Lower expression of *TNF*, Similar expression of *CXCL8*	Increased expression of proinflammatory cytokines	Irwin and Cole (26)

Moreover, our data set confirmed that *IL1B* may play a prominent role in the regulation of stress-induced immune responses fitting well with previous observations recorded for mammals (26) and fish (70). On the basis of those data, we hypothesize that the cytokines *IL1B, CXCL8*, and *TNF* may plausibly be responsible for the expression of several further genes in whitefish under HD compared with MD conditions. As in salmonid fish, *IL1B* (71, 72), *CXCL8* (73, 74), and *TNF* (75) serve as key proinflammatory mediators after pathogen invasion (76–79) and stress (17, 80, 81). We chose this as a starting point for the *in vitro* stimulation of head kidney leukocytes with *A. salmonicida* to study the influence of crowding on the induction of cytokine expression. Unexpectedly, SD manipulations had different effects on these genes. No differences were observed in the expression of *CXCL8* between cells from whitefish kept at MD or HD. In contrast, the expression of *IL1B* and *TNF* was obviously biased by SD conditions, though not unidirectionally: while the mRNA level of *TNF* was lower in stimulated cells from HD fish, the *IL1B* mRNA level was higher in challenged cells from HD fish compared with cells from MD fish. A similar effect on the expression of *IL1B* has been observed after long-term stress in salmon (81), emphasizing once more the presumably important role of IL1 during stress responses. It should be considered that anti-inflammatory immunosuppressive effects, such as those mediated through the stress hormone cortisol (18), may have also biased the recorded levels of cytokine-encoding transcripts in head kidney leukocytes. This argues for future studies into the complex regulation of teleost immunity under stress conditions addressing the questions of (i) how the immune status of whitefish develops after prolonged exposure to (crowding) stress, (ii) whether an additional stressor such as temperature further enlarges the observed expression differences, (iii) how the transcriptome of myeloid cells circulating in stressed fish differs from that in fish under normal conditions, and (iv) whether the observed transcriptional responses eventually contribute to immunopathologic damages or to the immunosuppression of whitefish in case of infection, particularly viral infections, with relevance to whitefish health [*cf* (82)].

In conclusion, the present study describes the physiological features of maraena whitefish exposed to increased stocking densities, revealing similar observations as in mammalian models: first, the increased mobilization of myeloid cells in the bloodstream, and second, CTRA-like profiles of several immune pathways significantly overexpressed in the liver and kidney of density-stressed whitefish (Table 5). These findings allow the conclusion that cost-intensive proinflammatory immune mechanisms are acutely activated in maraena whitefish as a consequence of crowding stress. At the same time, such adverse husbandry conditions potentially reduce antiviral defense capacities and activate several energy-supplying metabolic pathways. Thus, our data also point to the careful reconsideration of species-specific welfare conditions in aquaculture.

## Author Contributions

TG, BK, AR, and TK designed research; TK and BK performed immunological assays; AR, TG, SA, and MN performed gene profiling assays; TK, AR, MN, and SA, analyzed data; and TK and AR wrote the paper.

## Conflict of Interest Statement

The authors declare that the research was conducted in the absence of any commercial or financial relationships that could be construed as a potential conflict of interest. The reviewer AT and handling Editor declared their shared affiliation, and the handling Editor states that the process nevertheless met the standards of a fair and objective review.

## Abbreviations

CTRA, conserved transcriptional response to adversity; DE, differentially expressed; ED, elevated density; EDTA, ethylenediaminetetraacetic acid; EIF4E, eukaryotic translation initiation factor 4E; FDPS, farnesyl diphosphate synthase; FITC, fluorescein isothiocyanate; HD, high density; HSP, heat-shock protein; IPA, Ingenuity Pathway Analysis; LD, low density; MD, moderate density; MAb, monoclonal antibodies; PBL, peripheral blood leukocyte; PBS, phosphate-buffered saline; qPCR, quantitative real-time polymerase chain reaction; SD, stocking density; SSI, spleno-somatic index.
